# Spatial Pattern of Spring Mesozooplankton in the Marginal Ice Zone (Northern Barents Sea)

**DOI:** 10.3390/ani16081213

**Published:** 2026-04-16

**Authors:** Vladimir G. Dvoretsky, Alexander G. Dvoretsky

**Affiliations:** Murmansk Marine Biological Institute of the Russian Academy of Sciences (MMBI RAS), Murmansk 183038, Russia

**Keywords:** zooplankton, high-Arctic shelf, marginal ice zone, redundancy analysis

## Abstract

The effects of environmental factors on zooplankton within the marginal ice zone (MIZ) of the Barents Sea remain poorly understood. To address this knowledge gap, we investigated mesozooplankton communities across the central, northern, and northeastern regions in April 2016. Abundance and biomass ranged from 90 to 997 individuals m^−3^ and from 1.1 to 48.6 mg dry mass m^−3^ (0.3 to 13.6 g dry mass m^−2^), respectively. *Oithona similis* was the most abundant taxon, while calanoid copepods, including *Calanus* spp., *Metridia longa*, and *Pseudocalanus* spp., dominated total biomass. The spatial distribution of mesozooplankton communities was closely linked to the physical properties of water masses. Cluster analysis identified two distinct assemblages associated with Polar Front Water and Arctic Water. Redundancy analysis and generalized linear models identified temperature, mean salinity, mean chlorophyll a concentration, and sea ice concentration as significant predictors of abundance and biomass. The dominance of older life stages within major copepod taxa indicated a winter status for the mesozooplankton community. Regional and temporal comparisons of mesozooplankton biomass with prior May–June data from central and northwestern areas highlighted higher productivity in the northern and northeastern MIZ. This increase is potentially related to the warming trends observed in the Arctic since the 2000s. Our research provides novel insights into Arctic marine zooplankton assemblages and serves as a valuable baseline for future ecological monitoring and modeling of the Barents Sea ecosystem in the context of global climate change.

## Simple Summary

The influence of environmental factors on zooplankton in the marginal ice zone of the Barents Sea is not well understood. To fill this gap, we studied mesozooplankton communities in April 2016 across different regions. The most common species was *Oithona similis*, while larger copepods like *Calanus* spp. made up most of the biomass. The distribution of these zooplankton taxa was closely related to the water properties. We identified two main groups linked to different water types. Key factors affecting their abundance and biomass included temperature, salinity, chlorophyll *a* levels, and sea ice concentration. The presence of older copepods suggested they were in a winter state. Compared to past data, we noticed higher productivity in northern and northeastern areas, likely due to warming in the Arctic since the 2000s. This research helps understand Arctic zooplankton and provides a baseline for future studies on climate change impacts in the Barents Sea.

## 1. Introduction

Arctic marine environments are distinguished by pronounced seasonal variability in light availability, thermal conditions, and primary production [1,2]. The annually fluctuating sea-ice cover represents an additional key driver structuring Arctic marine ecosystems. In the Barents Sea, ice conditions are governed by the combined influence of Atlantic and Arctic water masses and by regional atmospheric forcing [3]. Since approximately 2000, during comparatively warm years, winter sea ice has been largely confined to the northern Barents Sea, whereas in colder years, ice also extends into the eastern and southeastern sectors. The maximum ice extent typically occurs in April and varies between 35 and 85%, with a mean value of 61% [3]. Consequently, the spatial extent and position of the Marginal Ice Zone (MIZ) in the Barents Sea show pronounced seasonal and interannual variability [4]. Arctic air and ocean temperatures have been increasing at a rate substantially exceeding the global mean, with estimates suggesting an amplification factor of at least 2–3 [5]. This rapid warming is linked to multiple interacting processes, including enhanced heat uptake by the Arctic Ocean, sea-ice decline, elevated near-surface air temperatures, and shifts in the circulation of major water masses, all of which propagate climate impacts across the Northern Hemisphere [6]. Projections indicate that Arctic warming will continue, reinforcing its status as a major global concern because of the region’s central role in modulating the Earth’s climate system [7].

The Barents Sea is an extensive shelf sea situated between the Arctic Ocean to the north and the Norwegian Sea to the southwest. Warm, saline Atlantic Water enters from the Atlantic Ocean and penetrates into central parts of the basin [8], while the northern Barents Sea is strongly affected by the inflow of cold Arctic waters [4]. This region sustains a rich and diverse marine biota and constitutes a critical fishing ground for several countries. Principal commercial resources include cod, haddock, capelin, herring, red king crab, snow crab, shrimp, and scallops [9,10,11,12]. The productivity, population dynamics, and distribution of these taxa are tightly coupled to the functioning of both pelagic and benthic components of the ecosystem [3].

A persistent warming signal has been documented in the Barents Sea, commonly termed Atlantification. This process reflects the increasing dominance of warmer, more saline Atlantic Water, with profound implications for the regional hydrography and the entire marine ecosystem [13,14]. Warm and cold years are classified based on temperature anomalies recorded along a standard transect in the Barents Sea. Specifically, in the Kola Section (69°30′–78°30′ N, 33°30′ E), positive temperature anomalies indicate warm years, while negative anomalies denote cold years [3]. Intensified surface-layer warming has driven a retreat of winter sea ice and the near-complete loss of summer ice cover. The reduction in winter ice inhibits the re-establishment of the cold, low-salinity surface layer generated by summer meltwater, thereby weakening vertical stratification and promoting enhanced mixing with underlying Atlantic Water—an oceanographic feature that is particularly characteristic of the Barents Sea [15,16]. Atlantification has led to substantial reorganization of the pelagic ecosystem. Shifts in fish migration routes have altered community composition and displaced many species and functional groups toward higher latitudes, which in turn has induced northward shifts in the distributions of seabirds and marine mammals [17]. Phytoplankton bloom zones have migrated approximately 200–300 nautical miles northward relative to their positions in the late twentieth century [18]. Over the last two decades, chlorophyll *a* concentrations and overall primary production have increased [19,20], and extensive blooms of coccolithophores such as *Emiliania huxleyi* have become typical during summer and autumn [21]. In contrast, glacial species and sympagic (ice-associated) organisms have declined as a consequence of diminishing sea-ice cover [19,22].

Marginal ice zones are widely recognized as hotspots of biological production [23,24]. In the Barents Sea, the MIZ is situated north of the Polar Front within Arctic Water masses [25]. During anomalously warm years, the ice edge retreats farther north, and primary production is enhanced compared with colder years [26]. Falk-Petersen et al. [27] identified three principal mechanisms underpinning high plankton production in the Barents Sea MIZ: (1) elevated primary production linked to the position and seasonal retreat of the ice edge; (2) advection of *Calanus finmarchicus* from the Norwegian Sea; and (3) transport of ice-associated fauna via the Transpolar Drift from the Arctic Ocean.

Zooplankton constitute key secondary producers and form a critical trophic link between phytoplankton and higher consumers, including fish, seabirds, and marine mammals [1,3,28]. Investigations of mesozooplankton in the Barents Sea MIZ have largely been confined to late spring and summer and have focused primarily on taxonomic composition, spatial distribution, and life-history traits of dominant taxa [10,11,25,29,30,31,32,33,34,35]. Furthermore, most previous studies have concentrated on the sector west of 40° E, leaving the eastern MIZ essentially unstudied in terms of zooplankton. Information on late-winter and early-spring conditions in this environment is also very limited [36,37].

Climate warming and the ongoing Atlantification have been shown to substantially reshape zooplankton assemblages in both the Barents Sea and the wider Arctic Ocean. Increased inflow of Atlantic Water and a progressively northward extension of boreal zooplankton species have been reported, signaling potential future changes in the relative abundance and community structure of Arctic versus Atlantic taxa in the northern Barents Sea [38]. Sea ice has emerged as a pivotal determinant of zooplankton community structure and productivity. Its influence is exerted directly, by providing physical habitat and supporting microalgal cryoflora that serve as an important food source for ice-associated and glacial species, and indirectly, via its role in shaping water column stratification, light conditions, and circulation [39]. Reductions in sea-ice cover and associated shifts in hydrographic conditions modify habitat suitability for species that inhabit or depend on the ice edge. Recent work has emphasized that sea ice is crucial for the persistence of large Arctic copepods, offering refuge from predators and thereby modulating trophic energy transfer and productivity on Arctic shelves [40]. In the northern Barents Sea, hyperiid amphipod abundance has been tightly linked to sea-ice conditions; declines in the biomass of *Themisto libellula* during years with reduced ice concentrations illustrate the pronounced sensitivity of zooplankton populations to sea-ice loss [37]. Seasonal changes in the abundance and composition of major zooplankton groups also reflect environmental variability. In the Fram Strait, sea-ice cover has been identified as one of the dominant drivers of zooplankton population dynamics [41]. Nonetheless, the magnitude and direction of sea-ice effects likely vary seasonally, and considerable uncertainty persists regarding its influence across different periods of the year [16]. Given the strong seasonality of zooplankton communities, it is reasonable to expect that their responses to sea-ice variability also exhibit pronounced seasonal differences.

The present study aims to characterize the distribution, taxonomic diversity, and spatial variability of mesozooplankton within the Barents Sea MIZ and to elucidate how these patterns relate to environmental gradients. We hypothesize that zooplankton abundance is closely and directly modulated by regional sea-ice conditions. In addition, we seek to assess how different water masses affect total zooplankton abundance and biomass and to evaluate our findings in the context of earlier estimates. The results are expected to enhance current understanding of the structure and functioning of high-Arctic pelagic ecosystems. Moreover, the dataset is intended to provide a baseline for future investigations of sea-ice-influenced marine systems.

## 2. Materials and Methods

### 2.1. Sampling and Laboratory Analyses

**Figure 1:**
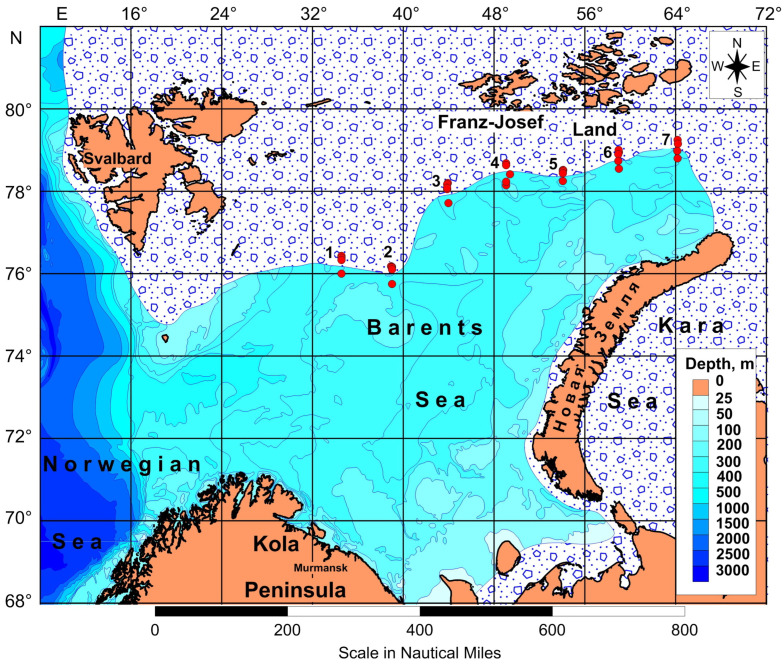
Location of sampling stations along the ice edge in the Barents Sea in April 2016. Data on sea ice cover in April 2016 were retrieved from the National Snow and Ice Data Center (https://nsidc.org; accessed on 18 May 2018) and Arctic and Antarctic Research Institute, Center of Ice and Hydrometeorological information (https://data.aari.ru; accessed on 25 July 2025).

Zooplankton sampling was carried out in the northern Barents Sea during an expedition of RV “Dalnie Zelentsy” in April 2016 (Figure 1, Table S1). The sampling took place within the Marginal Ice Zone (MIZ), a transitional area characterized by floating ice situated between the ice edge and the open sea, extending approximately 30–50 km southward from the ice edge [42].

A total of 33 stations were visited along 7 transects across the ice edge (Figure 1). The distance from the ice edge was instantaneously measured during the sampling using the vessel sonar. Sea-ice conditions were assessed visually from the vessel over an area of 100 m^2^, which was photographed to estimate ice coverage [43]. At each station, vertical profiles of temperature and salinity were obtained using an SBE 19 plus SEACAT CTD profiler (Sea-Bird Scientific, Bellevue, Washington, DC, USA). Zooplankton were collected with a Juday net (mouth opening 0.1 m^2^, mesh size 180 μm) towed vertically from 10 to 15 m above the seabed to the surface at a speed of 0.8–1 m s^−1^. Filtered water volume was estimated as the product of wire out (tow length) and net mouth area [44]. Immediately after retrieval, samples were preserved in a 4% borax-buffered seawater–formaldehyde solution.

Prior to zooplankton sampling, water samples for chlorophyll *a* determinations were collected with 10 L OTE PVC bottles (Hydro-Bios, Altenholz, Germany) from the surface, pycnocline, and near-bottom layers. Chlorophyll *a* was retained on Vladiopor filters (pore size 0.45 μm) (Science-Technical Center Vladiopor, Vladimir, Russia), which were stored at −22 °C until analysis. In the laboratory, chlorophyll *a* concentrations were measured using a Nicolett Evolution 500 fluorometer (Spectronic Unicam, Cambridge, UK) following Lorenzen and Jeffrey [45].

Zooplankton specimens were identified and enumerated under a stereomicroscope equipped with an ocular micrometer using standard protocols [44]. Most copepod taxa were resolved to species or genus level, and copepodite stages were determined for large, efficiently sampled species (*Calanus finmarchicus*, *C. glacialis*, *C. hyperboreus*, *Metridia longa*, and *Pseudocalanus minutus*). Discrimination among *Calanus* species was based on prosome length measurements [46,47]. Abundance was expressed as individuals per cubic meter (ind. m^−3^). Mesozooplankton biomass was estimated from abundance data using published species-specific dry weight values [48] and length–mass regression equations [49,50,51]. Wet mass was converted to dry mass (DM) using conversion factors of 0.04 for gelatinous zooplankton and 0.2 for other groups [44].

### 2.2. Data Processing

Zooplankton diversity was quantified in terms of species richness, the Shannon diversity index, and evenness. A similarity matrix for the seven transects was generated based on the Bray–Curtis similarity coefficient applied to mean zooplankton abundance [52]. This matrix was subjected to hierarchical agglomerative clustering using the group-average linkage method. All clustering procedures were carried out with PRIMER v5 [53]. To moderate the influence of numerically dominant taxa, zooplankton abundances were square-root-transformed prior to analysis. The robustness of the resulting clusters was evaluated with analysis of similarity (ANOSIM). Differences in the abundance of common mesozooplankton taxa among groups were tested using one-way ANOVA or, when parametric assumptions were not met, the Kruskal–Wallis one-way ANOVA on ranks [54]. Species contributing most strongly to within-cluster similarity were identified using SIMPER analysis. All multivariate and univariate statistical analyses were conducted in PRIMER version 5.2.3. To compare environmental and biological properties among station groups defined by cluster analysis, a one-way PERMANOVA was performed. Subsequently, an indicator species analysis was applied to identify taxa that best characterized individual clusters. This approach considers both the relative abundance and frequency of each taxon within a cluster, yielding an indicator value (IndVal) ranging from 0 to 1, where values closer to 1 indicate high specificity and fidelity to that cluster. Only indicator taxa with statistically significant IndVal scores were retained for interpretation [55].

Biological (abundance of dominant zooplankton taxa) and environmental variables were transformed using lg(x + 1) to improve normality prior to ordination analyses. Multicollinearity among environmental predictors was examined via variance inflation factor analysis (VIF > 5) in Canoco 4.5, and variables exhibiting strong collinearity were removed from subsequent models. Latitude, longitude, integrated chlorophyll *a*, mean temperature, surface salinity, and bottom salinity were excluded on this basis. Data normality was tested using the Shapiro–Wilk test while homogeneity of variances was assessed with a modified Levene’s test using PAST version 3.22 [56].

Detrended correspondence analysis (DCA) indicated gradient lengths between 0.377 and 0.931, justifying the use of redundancy analysis (RDA) based on approximately linear species–environment relationships [57]. RDA was applied to the zooplankton species abundance matrix. As a constrained ordination technique, RDA enables the derivation of synthetic environmental gradients directly from ecological data [58]. Ordination axes are constructed as linear combinations of the measured environmental variables. Nine environmental parameters were included in the final RDA: surface temperature, bottom temperature, mean salinity, surface chlorophyll *a*, bottom chlorophyll *a*, mean chlorophyll *a*, sampling depth, sea-ice concentration, and distance from the ice edge. In the ordination diagrams, arrow lengths for environmental variables are proportional to their explanatory importance [59], facilitating direct visualization of relationships between zooplankton community structure and environmental gradients. The overall significance of the species–environment relationship was evaluated with a Monte Carlo permutation test (999 permutations) applied to the full RDA model [57]. DCA and RDA were performed with Canoco 4.5. To complement the ordination, generalized linear models (GLMs) were fitted to quantify the dependence of abundant zooplankton taxa (those occurring at more than 90% of stations) on environmental variables. All response and predictor data were lg(x + 1)-transformed to meet normality assumptions, and GLMs were specified with an identity link and normal error distribution. Statistical significance was set at *p* < 0.05.

Descriptive statistics (ranges and means with standard errors) for oceanographic and biological variables were calculated using PAST version 3.22.

## 3. Results

### 3.1. Environmental Conditions

Transects 1 and 2 were sampled during the onset of stratification, with the thermocline located at a depth of 10–20 m from the surface. Surface temperature ranged between 1 °C and −1 °C (Table 1).

Transects 3 and 4 demonstrated low thermal stratification with a colder (<−1 °C) upper 30–70 m layer. Transects 5 and 6 were occupied by cold water < −1 °C from the surface to a depth of 150–200 m. The easternmost transect was characterized by definite stratification with warm waters (>0 °C) located below 70 m. Salinity was stable across the study area with mean value of >34.75 (Table 1).

The ice conditions varied from dense first year pack-ice with large ice floes to first-year ice with small ice floes. Floating ice was observed at all stations, with the highest concentrations recorded in the vicinity of the ice edge (Figure 1, Table S1).

Chlorophyll *a* concentrations were low, reaching maximum values in the surface layer (Table 2). Transect 2 was characterized by the highest chlorophyll *a* concentrations while the minimum was found in transect 3 (Table 1). Integrated chlorophyll *a* concentrations varied from 52 to 88 mg m^−2^ (Table 1).

### 3.2. Mesozooplankton Composition and Diversity

A total of 52 species or higher taxonomical categories were identified in the samples (Table 2).

The dominant zooplankton group was the class Copepoda, which was represented by 16 species; copepod nauplii, *Pseudocalanus* spp. stages I–IV, and *Paraeuchaeta* spp. stages I–IV were not identified to species level due to the morphological indistinguishability of their early developmental stages. 10 taxa were common in the study area (frequency of occurrence > 67%), including *Calanus finmarchicus*, *Calanus glacialis*, Copepoda nauplii, *Microcalanus pusillus*, *Microcalanus pygmaeus*, *Oithona similis*, *Pseudocalanus minutus*, *Themisto* spp. (in 100% of samples), *Oikopleura vanhoeffeni* (in 97% of samples), *Metridia longa* (in 90% of samples), and *Parasagitta elegans* (in 87% of samples). The number of taxa recorded ranged from 18 (St. 9) to 35 (St. 32). The analysis of diversity (Shannon index) revealed that zooplankton in transects 1 and 7 were more diverse than in the other transects (Table 2). A total of 19 sampling stations exhibited evenness values greater than 0.5, indicating a higher level of species distribution uniformity. Conversely, the remaining 14 stations recorded evenness values below 0.5. The highest values of evenness were found in transect 1 (Table 2).

### 3.3. Mesozooplankton Abundance and Biomass

Total mesozooplankton abundance varied by one order of magnitude among the sampling stations, ranging from a minimum of 90 ind. m^−3^ at St. 16 (transect 4) to a maximum of 997 ind. m^−3^ at St. 25 (transect 6). The mean total abundance (±standard error) for all stations was 489 ± 40 ind. m^−3^. Copepods comprised 87–99% (97 ± 1%) of the total zooplankton abundance. The second most abundant group was appendicularians, which accounted for 0–9% (2.0 ± 0.3%, 0–47 ind. m^−3^). The third most abundant group comprised amphipods (0–10%, 0.5 ± 0.2%, 0–22 ind. m^−3^) and meroplankton (0–4%, 0.5 ± 0.3%, 0–12 ind. m^−3^). Total mesozooplankton biomass ranged from 1.1 to 48.6 mgDM m^−3^, with a mean of 21.8 ± 2.4 mgDM m^−3^. Copepods constituted 46–99% (90 ± 2%) of the total mesozooplankton biomass. *Calanus* spp., *Metridia longa*, and *Pseudocalanus* spp. contributed most of the total mesozooplankton biomass (86 ± 11%). The integrated mesozooplankton biomass ranged among stations from 0.3 to 13.6 gDM m^−2^, averaging 5.7 ± 0.7 gDM m^−2^. Mean abundance and biomass values within the seven MIZ areas are shown in Table 2.

### 3.4. Mesozooplankton Community Structure

**Figure 2:**
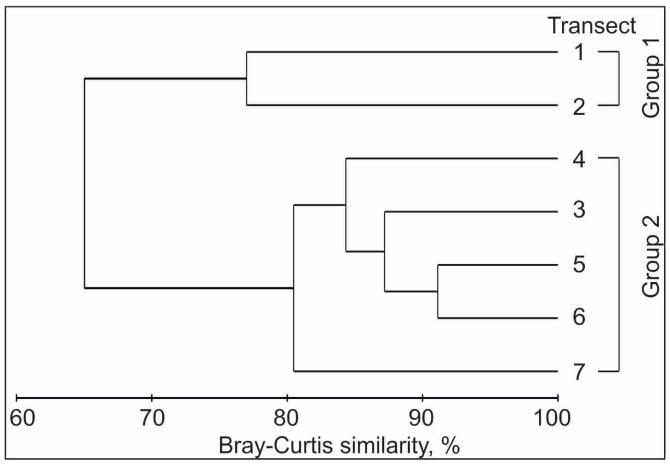
Dendrogram produced by the clustering of the 7 transects sampled in April 2016 in the Barents Sea based on the abundance of mesozooplankton taxa.

Analysis of similarities of mesozooplankton communities based on abundance indicated that two groups were significantly different in taxonomic composition (ANOSIM, global R = 1.0; *p* < 0.001, Figure 2).

Abundances of common zooplankton taxa were, in general, higher in Group 2, while temperatures were higher in Group 1 (Table 3).

The SIMPER test showed that the taxa contributing the most to the separation of the two groups of stations were *Oithona similis* (42% of the dissimilarity), *Calanus glacialis* (13%), *Pseudocalanus* spp. I–IV (12%), *Metridia longa* (9%), and *Pseudocalanus minutus* (6%). The IndVal analysis identified several indicator species for two groups (Table 4).

Six and ten taxa had significant IndVal in groups 1 and 2, respectively (Table 4). PERMANOVA tests found significant differences between two clusters based on both the environmental dataset (F = 61.25, *p* < 0.001) and on zooplankton abundance (F = 19.34, *p* < 0.001).

### 3.5. Stage Structure of Common Copepod Taxa

**Figure 3:**
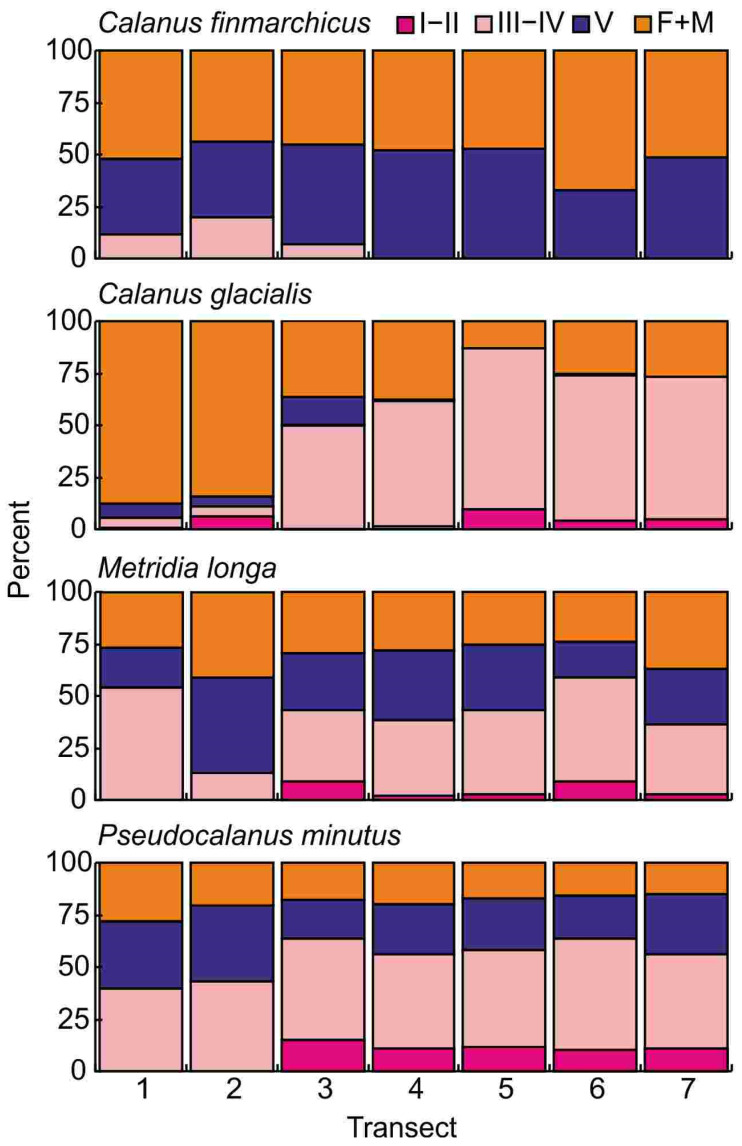
Relative abundance of common copepod taxa in the Barents Sea in April 2016. I–V—copepodites I–V, F—female, M—male.

The population of *Calanus finmarchicus* was dominated by late copepodite stages CV and by females. Together these accounted for 80–100% (94 ± 3%) of the individuals in all transects (Figure 3).

The relative stage composition of the population of *Calanus glacialis* changed from east to west (Figure 3). The contribution of stages CV and females decreased from 84 to 87% in transects 1–2 to 13–27% in transects 5–7 (Figure 3). Copepodites CI were only recorded in transect 2. Copepodites CII were absent in transect 3, while in other transects their proportion varied from 1 to 10%. The maximum concentrations of CIII–IV stages were found in transects 5 and 6 (Figure 3). The sex ratio (F:M) varied from 1:18 to 1:52 in transects 4–7.

The age structure of the *Metridia longa* population was similar across the study area, with CIII–IV dominating except in transect 2 where CV copepodites were the most abundant (Figure 3). Young copepodites were recorded in transects 3–7 but they made up less than 9% of the population (Figure 3). Males were found in all transects. The sex ratio varied from 1:5 to 1:14.

The population of *Pseudocalanus minutus* was composed primarily of CIII–IV stages, which contributed 40–54% of the total species density. Copepodites CI–II were absent in transects 1–2. Their relative abundance ranged from 10 to 15% in the rest of the study area (Figure 3). The sex ratio varied from 1:6 to 1:31.

All copepodite stages except for CI were detected in the population of *Oithona similis* in our study area, but only older copepodites (IV–V) and adults were considered in the analysis because of the low capture efficiency of the net used for the smaller stages. Copepodites V were the most numerous (29–47%) in transects 1–6 while females prevailed in transect 7 (35%). The sex ratio varied from 1:7 to 1:50.

### 3.6. Relationship Between Environmental Variables and Distribution of Mesozooplankton

A Monte Carlo test of the F-ratio showed that eight environmental variables contributed significantly to explaining the distribution of common taxa, explaining 75% of the total zooplankton–environmental variability (Table 5 and Table 6).

**Figure 4:**
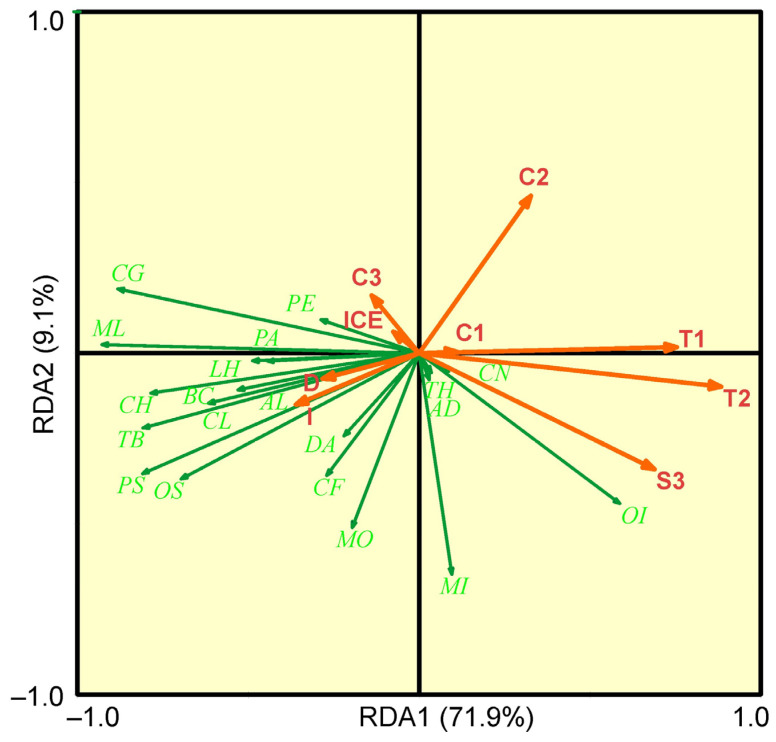
Ordination diagram of the redundancy analysis showing the common mesozooplankton taxa in relation to the environmental factors (T1—surface temperature, °C, T2—bottom temperature, °C, S3—mean salinity, C1—surface chlorophyll *a*, mg m^−3^, C2—bottom chlorophyll *a*, mg m^−3^, C3—mean chlorophyll *a*, mg m^−3^, D—sampling depth, m, ICE—distance from the ice edge, nautical miles, I—ice concentration, %). Taxa codes: CF—*Calanus finmarchicus*, CG—*Calanus glacialis*, CN—Copepoda nauplii, ML—*Metridia longa*, MI—*Microcalanus* spp., OS—*Oithona similis*, TB—*Triconia borealis*, PA—*Paraeuchaeta* spp., PS—*Pseudocalanus* spp., CL—*Clione limacina*, PE—*Parasagitta elegans*, TH—*Themisto abyssorum*, OI—*Oikopleura*, MO—*Mertensia ovum*, AL—*Aeginopsis laurentii*, AD—*Aglantha digitale*, DA—*Dimophyes arctica*, BC—*Beroe cucumis*.

The first two RDA axes accounted for 80.7% of the species–environment variability. The first axis was strongly positively correlated with surface and bottom temperatures and mean salinity, and slightly negatively correlated with mean chlorophyll a concentration, sampling depth, distance from ice cover, and sea ice concentration (Figure 4).

The second axis was positively correlated with bottom chlorophyll a concentration and negatively with mean salinity. Both axes were significant in the Monte Carlo test (*p* < 0.05). The right-hand side of the ordination diagram based on the first two axes (Figure 4) grouped samples with high temperature. Copepoda nauplii were more prevalent in relatively warm-water samples. Cold-water samples, associated primarily with negative temperatures and high sea ice concentration, were clustered in the left side of the diagram and were more strongly correlated with *Metridia longa*, *Calanus glacialis*, *C. hyperboreus*, *Pseudocalanus* spp., *Oithona similis*, *Triconia borealis* and *Clione limacine* (Figure 4).

The GLM analysis revealed that the abundances of the most frequent zooplankton taxa, including *Calanus glacialis*, Copepoda nauplii, *Metridia longa*, *Microcalanus* spp., *Oithona similis*, *Pseudocalanus* spp., and total zooplankton abundance showed significant negative correlations with temperature conditions (Table 7).

In contrast, the abundance of *Oikopleura* juveniles exhibited a positive relationship with increasing water temperature. Specifically, while *Calanus finmarchicus* abundance was negatively correlated with chlorophyll *a* concentration, an opposite trend was observed for Copepoda nauplii (Table 7). Moreover, total zooplankton abundance, *Calanus glacialis*, *Metridia longa*, *Oithona similis*, and *Pseudocalanus* spp. abundances all declined significantly with increasing mean salinity (Table 7). The distance from the ice edge was found to be a significant driver only for *Oikopleura* juveniles, demonstrating a negative correlation (Table 7). Sea ice concentration had a pronounced positive effect on total zooplankton abundance as well as the abundances of *Calanus glacialis*, *Oithona similis*, and *Pseudocalanus* spp. (Table 7). Interestingly, depth of sampling did not significantly influence the structuring of the zooplankton assemblage.

## 4. Discussion

### 4.1. Environmental Conditions

Four principal water mass types are typically distinguished in the Barents Sea: Atlantic Water (AW; relatively warm and saline), Arctic Water (ArW; low salinity and sub-zero temperatures), Barents Sea Water (BSW; cold and saline), and Coastal Water, which exhibits a broad range of temperatures and salinities depending on location [3,4]. In 2016, the sea-ice edge was displaced north of its long-term median position, and ArW occupied a broad zone east of 40° E. Transects 3–6 in our dataset were characterized by cold waters with temperatures below 0 °C and can therefore be classified as ArW transects. Transects 1–2 lay within the Polar Front region that separates warm, saline AW from colder, fresher Arctic waters and are thus categorized as Polar Front transects; hydrographic properties there were close to those of BSW. The easternmost sector (transect 7) was influenced by a two-layer water mass structure, with ArW in the upper 80 m and underlying BSW below this depth. This hydrographic configuration reflects the regional circulation in the northern Barents Sea [4], whereby cold Arctic waters enter the Barents Sea between Svalbard and Franz Josef Land and between Novaya Zemlya and Franz Josef Land, while warm Atlantic inflow from the Norwegian Sea splits into a northward and an eastward branch [1,16].

Comparison of observed water mass characteristics with climatological data showed that mean temperatures in our survey were similar to, or slightly above, long-term averages in the surface layer (−1 to −2 °C), but markedly higher at the seabed, where typical long-term means range from −1 to 0 °C [42]. This pattern is consistent with the sustained warming documented in the Barents Sea since the early 2000s [13,14,15]. The strength of Atlantic inflow into the Barents Sea is known to increase during positive phases of the North Atlantic Oscillation (NAO) and to weaken when the NAO index is low [60]. As a result, Barents Sea thermal conditions tend to co-vary with the NAO, with higher temperatures typically associated with positive NAO phases [8,61].

In the Barents Sea, phytoplankton growth near the ice edge generally initiates in late April, whereas in permanently ice-free sectors, the main spring bloom typically occurs during May–June [2]. Bloom onset is governed by increasing surface irradiance in combination with meltwater-induced stabilization of the upper water column [1]. Earlier studies have reported chlorophyll *a* concentrations ranging from 0.5 to 14 mg m^−3^ in the Barents Sea MIZ during May–June [25,26]. For instance, a pronounced bloom was observed in May 1993 in a strongly ice-covered and stratified Arctic zone of the central Barents Sea, with chlorophyll *a* exceeding 4 mg m^−3^ [26]. In June 1995, three successional stages of phytoplankton development (pre-bloom, early bloom, and bloom) were identified at ice stations in the northwestern Barents Sea, while a nearby open-ocean station exhibited post-bloom conditions [25]. Comparable patterns were found in May 1999 in the northern Barents Sea: pre-bloom conditions corresponded to integrated chlorophyll *a* of 30.4–68.2 mg m^−2^, bloom conditions to 162.1–195.8 mg m^−2^, and post-bloom conditions to 280.4–293.9 mg m^−2^ [36]. In other high-Arctic regions, including Svalbard waters and the northwestern Barents Sea, chlorophyll *a* concentrations are typically low in April–early May, reaching only 0.2–0.5 mg m^−3^ [20,62] or about 0.7–100 mg m^−2^ [38].

In our study, phytoplankton biomass was confined mainly to the upper 20 m, and chlorophyll *a* concentrations were uniformly low, indicative of a pre-bloom phase throughout most of the study region. A weak signature of an early bloom was detected only at stations close to the ice edge in transects 6–7, in agreement with the established conceptual model of spring phytoplankton development in the northern Barents Sea [28].

### 4.2. Mesozooplankton Composition and Diversity

The most recent comprehensive checklist of Arctic zooplankton reports 145 free-living invertebrate species for the northern Barents Sea [63]. The majority of taxa encountered in this survey have previously been documented from Arctic Waters of the Barents Sea, where copepods typically dominate the zooplankton community [25,30,33,34,64,65,66]. The April 2016 zooplankton assemblage can be broadly separated into three faunal groups. The first group comprises true Arctic species, including *Aeginopsis laurentii*, *Dimophyes arctica*, *Calanus glacialis*, *C. hyperboreus*, *Metridia longa*, *Themisto libellula*, *Clione limacina*, *Limacina helicina*, and *Gaetanus tenuispinus*. The second group consists of broadly distributed cosmopolitan taxa such as *Oithona similis*, *Pseudocalanus* spp., and *Microcalanus*. The third group includes boreal and warm-temperate species, for example *Calanus finmarchicus* and *Oithona atlantica*. Taxa from the first two groups were widespread across the study area and were primarily associated with ArW. Boreal taxa were recorded throughout all oceanographic transects in the northern Barents Sea, a pattern attributable to two primary factors. First, the northward range expansion of warm-water species is associated with an increased influx of Atlantic Water—a trend that has markedly intensified since the early 21st century. This phenomenon, referred to as “borealization”, has also been observed in other components of the marine ecosystem, including phytoplankton and fish [8,14,18,41]. The second key factor contributing to this pattern is pan-Arctic warming [7,13], which has resulted in elevated water temperatures. This warming facilitates the successful reproduction and development of Atlantic species well beyond their typical distribution ranges [10,11,19,38]. However, disentangling the relative contributions of these factors to zooplankton dynamics remains a challenge. It is likely that their synergistic effects have played a significant role. We posit that a long-term regime shift may be the critical factor driving the notably high abundance of boreal species observed in our study.

Our results demonstrate high mesozooplankton species richness and elevated Shannon diversity. The diversity estimates exceeded those reported for May 1999 [30] in the MIZ and were comparable to, or higher than, values documented during some summer periods in the free-ice Barents Sea [11,67]. This pattern can be primarily attributed to a reduced dominance of single taxa. In open-ocean summer conditions, it is common for one or two species to dominate the zooplankton community; for instance, the combined relative abundance of *Oithona similis* and *Calanus finmarchicus* can exceed 70% in the southern and central Barents Sea [10,11,66,68,69]. The low diversity reported by Blachowiak-Samolyk et al. [30] for the MIZ in May 1999 was linked to very high numbers of *O. similis*. In contrast, the high evenness observed in April 2016 reflects both greater species richness and a more equitable distribution of individuals among taxa. Therefore, our research indicates that waters adjacent to the ice edge exhibited greater diversity metrics.

Our analyses further suggest that the highest species richness and diversity occurred in waters adjacent to the ice edge. This pattern indicates a positive effect of the ice-edge environment on zooplankton community structure, abundance, and diversity. Ice-edge regions function as dynamic frontal zones that separate distinct water masses and are frequently characterized by elevated biomass and taxonomic diversity. These conditions are supported by enhanced primary production (driven by nutrient supply and stratification) and by a variety of microhabitats favorable to different zooplankton life strategies. Comparable trends—higher zooplankton diversity and biomass in frontal and ice-edge zones—have been documented for spring, summer, and autumn assemblages elsewhere in the Barents Sea, underscoring the ecological importance of such interfaces [11,31,32,33,34].

### 4.3. Mesozooplankton Abundance and Biomass

In our survey, relatively high mesozooplankton abundances (>400 ind. m^−3^) were recorded in ArW, whereas Polar Front waters exhibited lower mean densities. Pronounced patchiness in zooplankton distribution is a well-known feature of Arctic marine systems [10,11,31,33,34]. In general, such spatial heterogeneity reflects the combined effects of hydrographic structure, mesoscale circulation, advection from neighboring areas, and seasonal progression of zooplankton populations [70]. In the present case, differences in abundance appear to be primarily linked to water mass characteristics. During May 1999, substantial spatial variation in mesozooplankton density was observed in the northwestern Barents Sea MIZ, with total zooplankton concentrations at individual stations ranging from 899 ind. m^−3^ in open waters to 6 815 ind. m^−3^ near the ice edge [30,71]. In June 1995, total zooplankton abundance in the northern Barents Sea ranged from 15,705 to 94,128 ind. m^−2^ [25]. Abundances in our Arctic transects 3–7 were higher than those reported for June but lower than May estimates. Copepods dominated at all stations, consistent with earlier observations from the Barents Sea MIZ during spring and summer [25,29,71], as well as from Atlantic and coastal regions of the Barents Sea [11,32,66].

Total zooplankton biomass is widely regarded as one of the most reliable indicators of secondary plankton productivity in marine ecosystems [33,34,70]. While this metric is sensitive to stage-specific variations in body mass, the biochemical composition of different age groups of zooplankton, and seasonal fluctuations in population and size structure, it remains an effective tool for providing robust estimates of zooplankton production and carbon transfer efficiency within marine ecosystems [44]. In the Barents Sea, zooplankton biomass exhibits pronounced seasonal and interannual variability [19,65,72,73], driven by advective inputs and local environmental forcing [28]. For example, in the central Barents Sea during late spring/early summer 1979–1984, interannual means of zooplankton biomass ranged between 2 and 20 g DM m^−2^ [74], and seasonal changes in biomass may span an order of magnitude [68]. From 1989 to 2017, autumn surveys revealed strong year-to-year fluctuations in mesozooplankton biomass and an inverse relationship with capelin biomass; since the mid-2000s, mean autumn biomass has stabilized at approximately 6–8 g DM m^−2^ [19].

Our data indicate significant spatial variability in mesozooplankton biomass, with a distinct trend of increasing biomass from the central Barents Sea transects in the west toward the northern and northeastern sectors. This gradient likely reflects a combination of regional differences in the phenological stages of zooplankton communities, spatial variability in zooplankton size structure, and the influence of advected Arctic waters. Specifically, the higher biomass observed in the northern and northeastern transects may be attributed to greater abundances of large calanoid copepods (genus *Calanus*) compared to those in the western transects. Earlier work in the Barents Sea MIZ reported total mesozooplankton biomasses < 10 g wet mass m^−2^ (i.e., <2 g DM m^−2^) in May 1981 [75], and 1–5 g DM m^−2^ in March and May 1998 [76]. In the northwestern Barents Sea, large zooplankton biomass ranged from 0.01 to 0.25 g wet mass m^−3^ during March–May 2021 at stations with sea-ice concentrations of 71–94%, whereas in the central Barents Sea, where ice cover was lower (0–86%), biomass varied between 0.01 and 0.02 g wet mass m^−3^ [37]. In another study, mesozooplankton abundance and biomass at ice-covered stations in the northern Barents Sea during March–May were 88–453 × 10^3^ (mean 264 × 10^3^) ind. m^−2^ and 1.1–16.9 (mean 8.2) g DM m^−2^, respectively [38]. Hop et al. [77] reported total mesozooplankton abundances of 70–215 ind. m^−3^ in the upper 50 m of the ice-covered Arctic Ocean during March–April 2015. In Svalbard coastal waters, total zooplankton biomass was 0.5–1.9 g DM m^−2^ in May 2019–2020 [62].

Against this background, our biomass estimates for transects 1, 2, and 4 (1–4 g DM m^−2^) are broadly consistent with previous findings, whereas values in the remaining transects (8–11 g DM m^−2^) are notably higher, suggesting enhanced mesozooplankton productivity in the northeastern MIZ. This elevated productivity may be linked to the warming of the Barents Sea during the first decade of the twenty-first century; positive effects of increased water temperature on mesozooplankton biomass in the region have been reported previously [10,11,66,68,72]. An additional contributing factor may be reduced predation pressure from carnivorous zooplankton and planktivorous fishes in the northeastern sector [8,19].

### 4.4. Mesozooplankton Community Structure

The mesozooplankton communities identified in this study were broadly structured along gradients in sea surface temperature, which effectively distinguish the two principal water masses present. The two main assemblages revealed by cluster analysis were primarily differentiated by total zooplankton abundance and by the densities of dominant copepod species. Cluster 1 comprised stations in Polar Front waters and was characterized by comparatively low mesozooplankton abundance and biomass, whereas Cluster 2 encompassed stations influenced by Arctic Water. *Oithona similis* was the numerically dominant species at all stations, contributing 43–56% of total mesozooplankton abundance. This dominance is consistent with its known ubiquity in Arctic waters and its considerable physiological and morphological plasticity. The assemblage associated with Polar Front waters can be described as an Arctic–boreal community, distinguished by a greater contribution of Atlantic-affiliated species such as *Calanus finmarchicus* and *Oithona atlantica*. In contrast, the community in Arctic Water represents a more strictly Arctic fauna, with *Calanus glacialis* as the most abundant calanoid copepod. Strong coupling between zooplankton community structure and water mass characteristics has been repeatedly documented for the Barents Sea and neighboring Arctic regions [10,11,28,37,65,66,68,77], and our results corroborate this general pattern. Indicator species analysis further demonstrated that particular taxa were diagnostic of the Polar Front and Arctic Water domains. Warm-water and cosmopolitan forms such as common appendicularians and *Oithona atlantica* were associated with the frontal zone and warmer waters (Transects 1–2), whereas typical cold-water taxa including *Calanus glacialis*, *Calanus hyperboreus*, *Clione limacina*, *Themisto libellula*, and *Paraeuchaeta* spp. served as indicators of stations influenced by Arctic Water.

The elevated mesozooplankton abundance observed along transects 3–7 (Cluster 2, Arctic Water) likely reflects the combined influence of seasonal population development and advective processes. First, increased abundance and biomass in the eastern transects may represent a stage in the spring progression of the zooplankton community. Rapid increases in zooplankton standing stock over one to two weeks in spring have been reported in the southern Barents Sea [11,32,34,68], in Svalbard coastal waters [78], and in the White Sea [79]. Second, high abundances and biomasses may be linked to the inflow of cold Arctic waters, which transport populations of Arctic species such as *Calanus glacialis*, *C. hyperboreus*, and *Pseudocalanus minutus* [38,80], thereby enriching zooplankton communities near the ice edge. Because the northern and northeastern Barents Sea are more strongly influenced by this advective flux than central and western areas, these regions tend to support higher abundances and biomasses of cold-water taxa. Enhanced productivity of Arctic Water relative to Polar Front Water has likewise been described for macrozooplankton in the Barents Sea MIZ during late winter and spring [36].

### 4.5. Stage Structure of Common Copepod Taxa

Copepods of the genus *Calanus* are key components of Arctic zooplankton, typically contributing the bulk of mesozooplankton biomass [80]. *Metridia longa* and *Pseudocalanus minutus* are also major contributors to mesozooplankton abundance and biomass in the Barents Sea [10,11,25,33,34,65,66,76,81,82]. *Oithona similis* is generally the most numerous species in the region, often constituting more than 40–60% of total mesozooplankton abundance in various seasons [10,11,33,34,38].

Contrasting life-history strategies of the dominant copepod taxa during spring in the northern Barents Sea underlie the observed spatial and temporal variation in their abundance and stage structure. *Calanus finmarchicus* does not complete its life cycle in the northern Barents Sea and is regarded as an expatriate species in Arctic waters [10,11,29,33,34,80]. Our data support this interpretation: 80–100% of the *C. finmarchicus* population occurred as CV and adults (females), which are the principal overwintering stages. A similar stage composition, with dominance of CV, was reported east of Svalbard in May 1995 [25].

*Calanus glacialis* is a characteristic Arctic shelf species that reproduces in the central and northern Barents Sea [80]. It is known to have a life span of 1–3 years and to spawn before or during the onset of the spring bloom [83]. In this study, all developmental stages of *C. glacialis* were recorded, including nauplii, although early copepodite stages were relatively scarce. Polar Front waters were dominated by adults, in agreement with earlier observations from the northwestern Barents Sea MIZ in May [25,38,83]. In Arctic waters, CIV formed more than 30% of the population, suggesting that a large fraction of the population remained in a dormant state and that only a limited proportion had reproduced by April 2016. These results are consistent with previous findings indicating that *C. glacialis* egg production in the Barents Sea is closely synchronized with the phytoplankton spring bloom [84]. A dominance of *Calanus* spp. in total zooplankton biomass is also typical of other Arctic regions during the pre-bloom period [38,77]. *Pseudocalanus minutus* is regarded as an oceanic species that generally reproduces in May–June and completes an annual life cycle in Arctic waters [85], although spawning may occasionally commence before the main spring bloom [83]. Copepodite stages III–V of *Pseudocalanus* spp. are able to overwinter at stages III–V. In our material, copepodite stages III–V accounted for 68–79% of total *P. minutus* abundance, indicating that the bulk of the population was still overwintering in April 2016. In May 1995, later stages likewise dominated at all stations in the northwestern Barents Sea [25]. Similar patterns have been reported from Svalbard coastal waters, where older stages prevailed from November through March [85]. The presence of nauplii and early copepodites (CI–CII) in the northern and northeastern Barents Sea (transects 3–7) nonetheless indicates that some fraction of the *P. minutus* population initiated reproduction prior to the main spawning peak.

*Metridia longa* is an omnivorous species that inhabits intermediate layers in Arctic waters [32,82,86] and reproduces more or less continuously throughout the year [87]. Previous research in Svalbard waters has shown that *M. longa* has a spawning maximum in April–May [85,87]. In our study, early stages of *M. longa* (including nauplii) were present but constituted only 3–7% of the population, suggesting limited reproductive activity in April. This finding is consistent with earlier results from May 1995, when older stages of *M. longa* dominated and only a small fraction of young copepodites was present [25].

*Oithona similis* is a small cyclopoid species with an omnivorous detritivorous feeding strategy that is active year-round [35,88]. This species can spawn throughout the year and is capable of producing 1–2 main generations during the principal reproductive period [87,88]. In Svalbard waters, *O. similis* exhibits two main reproductive peaks, in June and August/September [85]. Our study documented early stages of *O. similis* in the samples, but they comprised only 5–9% of the total population. This may be related to the low capture efficiency of the net used. On the other hand, spawning activity of *O. similis* appeared to be low in April 2016.

Taken together, these results indicate that, in April 2016, the mesozooplankton community in the Barents Sea MIZ largely retained the characteristics of a winter assemblage. This conclusion is in line with the widely recognized coupling between zooplankton seasonality and phytoplankton development, and with the central role of food availability in regulating reproductive activity of many mesozooplankton species in Arctic ecosystems [10,11,28,33,34,68,70].

### 4.6. Relationship Between Environmental Variables and Distribution of Mesozooplankton

Redundancy analysis indicated that water temperature (surface and bottom), mean salinity, chlorophyll *a* concentration, and sea-ice concentration were key drivers of species composition and spatial variability in zooplankton abundance. Temperature and salinity represent fundamental descriptors of water mass properties. In this dataset, spatial variation in salinity was limited, implying that temperature was the main differentiating hydrographic feature among stations. Densities of *Calanus glacialis* and *C. hyperboreus* increased with decreasing temperature, higher sea-ice concentrations, and eastward position, highlighting their association with Arctic Water. In contrast, *Calanus finmarchicus* exhibited the opposite pattern, emphasizing its strong affinity for Atlantic-derived waters. Other abundant taxa, such as *Oithona similis*, *Metridia longa*, and *Pseudocalanus* spp., displayed pronounced associations with ice conditions, suggesting a potentially positive effect of sea ice on their development. This may reflect their feeding ecology: during periods of low phytoplankton biomass, these omnivorous taxa can exploit alternative food sources, including algal cryoflora and protozooplankton associated with sea ice [88].

The role of environmental variability in shaping zooplankton community structure in the Barents Sea has been extensively documented. Blachowiak-Samolyk et al. [30] showed that approximately 55% of mesozooplankton variability in the Barents Sea MIZ could be explained by depth layer, fluorescence, temperature, salinity, bottom depth, latitude, bloom stage, and ice concentration. Similarly, Søreide et al. [36] demonstrated that temperature and salinity were the primary explanatory variables for macrozooplankton variability in the central and northwestern Barents Sea during late winter and spring. In the northern Barents Sea, Van Engeland et al. [37] found that water temperature, salinity, depth, and chlorophyll *a* concentration collectively explained more than 45% of the variation in large zooplankton communities under ice-covered conditions. Comparable conclusions were reached for Kongsfjorden, where the same suite of physical and biological drivers strongly influenced zooplankton assemblage structure [77].

In the present study, generalized linear models corroborated the RDA results by confirming that temperature, salinity, and chlorophyll *a* concentration were among the most influential predictors of zooplankton distribution. Sampling depth and distance from the ice edge, by contrast, had no significant direct effect on zooplankton assemblages in early spring. These findings underscore the overriding importance of hydrographic conditions—and, by implication, of circulation patterns and water mass advection—in structuring mesozooplankton communities in the northern Barents Sea. Although the positive influence of ice-edge dynamics on plankton development in spring is well established and often linked to elevated chlorophyll *a* concentrations and enhanced microalgal production [11,38,77], no pronounced phytoplankton bloom was detected at the ice edge during our sampling. The absence of a bloom likely contributed to relatively modest zooplankton abundance and biomass, given the central role of bloom events in fueling zooplankton growth and reproduction.

Ice concentration emerged in our analyses as a significant environmental factor regulating zooplankton abundance in the pre-bloom period, in line with our initial hypothesis on the importance of ice conditions for zooplankton distribution. Numerous studies have similarly highlighted the central role of sea ice in determining zooplankton community composition, abundance, and biomass in Arctic ecosystems [39,41]. For instance, Wold et al. [38] showed that sea-ice cover is a major determinant of mesozooplankton assemblages in the northern Barents Sea. Sea ice also affects zooplankton indirectly by altering oceanographic properties such as temperature and salinity [39]. Moreover, it serves as habitat for sympagic fauna, including hyperiid amphipods and other ice-associated species that are especially concentrated near the ice edge, where they feed, grow, and reproduce. A range of taxa, including *Calanus glacialis*, *Calanus hyperboreus*, *Clione limacina*, and *Dimophyes arctica*, make extensive use of the ice-edge habitat at various stages of their life cycles, further illustrating the pivotal ecological role of sea ice in structuring high-Arctic pelagic communities.

## 5. Conclusions

Large-scale surveys aimed at clarifying the spatial distribution of zooplankton in the northern Barents Sea are crucial not only for enhancing our understanding of the Arctic marine ecosystem but also for environmental and biotic monitoring in light of climate-related changes, particularly those associated with global warming. Investigations of ice edge communities yield valuable insights into pelagic ecosystems and their productivity. Our study represents a significant advancement in this field, as it is the first to detail the mesozooplankton community in the marginal ice zone (MIZ) of the northeastern Barents Sea, while also contributing new data regarding the central and northern regions of this area. We identified clear distinctions in mesozooplankton composition between two primary water masses: Polar Front Water and Arctic Water, with elevated productivity observed in the more eastern regions. This pattern may be linked to the advection of cold-water species from the Arctic Ocean. We found that mesozooplankton abundance was significantly related to temperature, salinity, mean chlorophyll *a* concentration, and other hydrological parameters. The prevalence of late stages of common copepod taxa indicates that the mesozooplankton community is reflective of winter conditions, characterized by pre-bloom states and low phytoplankton biomass. Temporal comparisons of mesozooplankton biomass in the MIZ of the Barents Sea demonstrated that current values are greater than those recorded in May and June. This finding is noteworthy as it suggests a potential increase in zooplankton productivity during the early spring period. Our research significantly enriches the existing knowledge regarding the functioning, diversity, and composition of high-Arctic pelagic communities during the pre-bloom period, while also providing new perspectives on the role of sea ice as a critical factor influencing zooplankton assemblages. Given the evident fluctuations in climatic conditions and ongoing warming trends, it is reasonable to anticipate that further changes in the composition of pelagic fauna and total plankton stocks will occur. Monitoring and modeling these potential alterations will be imperative. The baseline data generated from our study are of great importance and applicability for these future efforts, as they provide essential context for understanding how environmental changes may impact Arctic marine ecosystems. Several critical issues persist in our understanding of Arctic pelagic ecosystems, necessitating further detailed investigation. In particular, research is needed to elucidate the vertical distribution and diel dynamics of zooplankton in the northern Barents Sea, as well as the functional diversity and production rates of zooplankton within the marginal ice zones. Additionally, it is important to assess the contribution of marine zooplankton to the carbon cycle in high Arctic regions. A systematic study of these aspects will significantly enhance our comprehension of the structure and functionality of Arctic pelagic ecosystems. Further research is essential to address these knowledge gaps.

## Figures and Tables

**Table 1:** Environmental conditions (T—temperature, °C; S—salinity; C—chlorophyll *a*, mg m^−3^, C′—integrated chlorophyll *a*, mg m^−2^) in the Barents Sea in April 2016.

Parameter	Transect 1 (St. 1)	Transect 2 (St. 5)	Transect 3 (St. 10)	Transect 4 (St. 15)	Transect 5 (St. 20)	Transect 6 (St. 25)	Transect 7 (St. 31)
Tsurface	0.9	−1.2	−0.9	−1.5	−1.8	−1.8	−1.3
Tbottom	2.1	1.9	0.4	−0.2	0.0	−0.6	1.1
Tmean	1.8	1.4	−0.3	−0.6	−1.1	−1.5	1.3
Ssurface	34.66	34.54	34.76	34.75	34.75	34.64	34.51
Sbottom	34.97	34.97	34.96	34.91	34.92	34.88	34.87
Smean	34.89	34.86	34.85	34.87	34.83	34.79	34.83
Csurface	0.36	0.93	0.34	0.36	0.49	0.77	0.75
Cbottom	0.14	0.15	0.08	0.21	0.08	0.14	0.11
Cmean	0.29	0.53	0.36	0.30	0.37	0.52	0.67
C′	59	88	66	27	71	69	64

**Table 2:** Mesozooplankton composition, mean abundance of taxa (individuals m^−3^), total abundance (individuals m^−3^), integrated abundance (10^3^ individuals m^−2^), evenness (E), Shannon index (H′), total biomass (mg dry mass m^−3^), integrated biomass (g dry mass m^−2^) in the Barents Sea in April 2016.

Taxon (Group)/Parameter	Transect						
	1	2	3	4	5	6	7
*Calanus finmarchicus*	14	5	18	3	13	8	8
*Calanus glacialis*	7	2	38	29	49	66	68
*Calanus hyperboreus*	-	-	1	1	8	8	7
*Chiridius obtusifrons*	-	-	-	-	-	-	<1
Copepoda nauplii	19	31	21	9	6	29	51
*Gaetanus tenuispinus*	-	-	<1	-	<1	<1	<1
*Harpacticus uniremis*	-	-	<1	<1	<1	-	<1
*Metridia longa*	5	<1	40	21	39	72	32
*Microcalanus pusillus*	3	4	4	2	1	2	1
*Microcalanus pygmaeus*	29	35	52	27	25	28	14
*Microsetella norvegica*	<1	<1	1	<1	<1	<1	<1
*Oithona atlantica*	2	3	1	1	<1	<1	2
*Oithona similis*	84	176	296	256	364	400	186
*Triconia borealis*	-	<1	1	2	5	5	3
*Paraeuchaeta* spp. I–IV	<1	<1	1	<1	<1	<1	<1
*Paraeuchaeta glacialis* V–VI	<1	-	-	<1	<1	<1	<1
*Pseudocalanus* spp. I–IV	6	13	66	39	83	82	24
*Pseudocalanus minutus* V–VI	8	20	38	36	56	45	18
*Scolecithricella minor*	-	-	-	-	-	-	<1
*Aeginopsis laurentii*	-	<1	<1	<1	<1	<1	<1
*Aglantha digitale*	<1	<1	<1	<1	<1	<1	<1
*Dimophyes arctica*	<1	<1	<1	<1	<1	<1	<1
*Euphysa flammea*	-	<1	<1	<1	<1	<1	<1
*Euphysa* spp. *juv.*	<1	<1	-	<1	-	<1	-
*Perigonimus yoldiaarcticae*	-	-	-	-	<1	-	-
Bivalvia juv.	-	<1	-	-	-	-	-
Echinopluteus larvae	-	<1	-	-	-	-	-
Gastropoda larvae	<1	2	1	<1	1	<1	<1
Ophiopluteus larvae	<1	<1	-	-	-	-	-
Polychaeta larvae	1	5	-	-	<1	<1	<1
*Chionoecetes opilio* larvae	-	-	-	-	<1	-	-
*Hyas* spp. zoea	-	<1	-	-	-	-	-
*Boroecia borealis*	-	-	-	-	-	<1	<1
Pisces larvae	-	-	-	-	-	-	<1
*Clione limacina* larvae	<1	<1	<1	<1	<1	1	<1
*Clione limacina*	-	<1	-	<1	<1	<1	<1
*Limacina helicina* larvae	<1	<1	<1	<1	<1	1	<1
*Eukrohnia hamata*	-	-	-	-	-	<1	<1
*Parasagitta elegans*	<1	<1	<1	<1	<1	<1	1
*Thysanoessa inermis*	<1	-	<1	<1	<1	<1	<1
*Thyssanoessa* spp. furcilii	<1	-	<1	-	-	-	-
*Hyperia galba*	-	-	<1	-	-	-	-
*Themisto abyssorum*	2	<1	<1	<1	-	<1	<1
*Themisto libellula*	-	-	-	<1	<1	<1	<1
*Themisto* juv.	3	<1	1	<1	<1	1	1
*Onisimus* spp.	-	-	-	-	-	-	-
*Fritillaria borealis*	4	12	-	-	-	-	-
*Oikopleura* juv.	5	9	1	1	1	1	<1
*Oikopleura vanhoeffeni*	-	1	1	<1	1	1	<1
*Beroe cucumis*	-	<1	<1	<1	<1	<1	<1
*Mertensia ovum*	<1	<1	<1	<1	<1	<1	<1
Sipuncula larvae	<1	<1	-	-	-	-	-
Foraminifera	-	-	-	<1	-	-	-
Total abundance	195 ± 13	318 ± 64	584 ± 54	429 ± 89	655 ± 75	753 ± 99	417 ± 52
Integrated abundance	48 ± 2	74 ± 14	170 ± 16	106 ± 24	175 ± 20	169 ± 29	132 ± 18
E	0.619	0.502	0.519	0.467	0.459	0.486	0.523
H′(log_e_)	1.859	1.603	1.700	1.464	1.547	1.609	1.789
H′(log_2_)	2.683	2.313	2.452	2.112	2.232	2.321	2.581
Total biomass	10 ± 4	4 ± 2	29 ± 3	17 ± 3	21 ± 4	36 ± 3	36 ± 5
Integrated biomass	2 ± 1	1 ± 1	9 ± 1	4 ± 1	6 ± 1	8 ± 1	11 ± 1

**Table 3:** Significant differences in mesozooplankton abundance and environmental conditions between two groups delineated by cluster analysis in the Barents Sea in April 2016. F—Fisher’s value, H—chi-square value.

Taxon/Parameter	Mean ± SE		One-Way ANOVA		Kruskal–Wallis Test	
	Group 1	Group 2	F	*p*	H	*p*
Abundance						
*Calanus glacialis*	4 ± 3	53 ± 6	-	-	17.673	<0.001
*Calanus hyperboreus*	<1	5 ± 1	-	-	16.014	<0.001
*Metridia longa*	2 ± 2	41 ± 5	20.36	<0.001	-	-
*Oithona similis*	135 ± 24	305 ± 25	15.15	<0.001	-	-
*Triconia borealis*	0.11 ± 0.05	3 ± 1	-	-	16.833	<0.001
*Paraeuchaeta*	0.09 ± 0.03	0.40 ± 0.07	-	-	10.197	<0.05
*Pseudocalanus* spp.	24 ± 6	100 ± 11	-	-	14.437	<0.001
*Clione limacina*	0.11 ± 0.08	0.48 ± 0.09	-	-	10.197	<0.05
*Oikopleura*	14 ± 3	3 ± 0	-	-	11.805	<0.05
*Beroe cucumis*	0.01 ± 0.01	0.04 ± 0.01	-	-	4.250	<0.05
Total	263 ± 40	578 ± 41	18.76	<0.001	-	-
Environmental conditions						
Tsurface	0.6 ± 0.4	−1.4 ± 0.1	42.37	<0.001	-	-
Tbottom	1.8 ± 0.1	0.0 ± 0.1	135.82	<0.001		
Tmean	1.6 ± 0.1	−0.6 ± 0.1	-	-	18.36	<0.001
Sbottom	34.96 ± 0.01	34.89 ± 0.02	-	-	11.26	<0.05
Smean	34.89 ± 0.01	34.83 ± 0.01	21.62	<0.001	-	-

**Table 4:** Indicator species according to IndVal analysis in the Barents Sea in April 2016.

Taxon	Group 1		Group 2	
	IndVal, %	*p*	IndVal, %	*p*
*Fritillaria borealis*	100.0	0.0001	0.0	1.0000
*Oikopleura* juv.	89.8	0.0001	9.3	1.0000
Polychaeta larvae	86.3	0.0002	0.8	1.0000
*Oithona atlantica*	72.9	0.0051	21.5	0.9941
*Microcalanus pusillus*	62.0	0.0188	38.0	0.9813
Gastropoda larvae	59.9	0.0374	15.3	0.9918
*Metridia longa*	3.8	1.0000	94.4	0.0001
*Triconia borealis*	1.5	1.0000	92.6	0.0001
*Calanus glacialis*	7.6	1.0000	92.4	0.0001
*Calanus hyperboreus*	0.0	1.0000	91.7	0.0001
*Pseudocalanus* spp.	14.0	1.0000	86.0	0.0001
*Paraeuchaeta* spp.	10.0	1.0000	82.1	0.0001
*Clione limacina* larvae	6.2	0.9995	74.8	0.0006
*Pseudocalanus minutus*	26.8	0.9998	73.2	0.0003
*Limacina helicina* larvae	9.1	0.9931	69.6	0.0070
*Oithona similis*	30.7	0.9999	69.4	0.0002
*Themisto libellula*	0.0	1.0000	66.7	0.0002

**Table 5:** Results of the forward selection procedure of environmental variables in the RDA explaining variability in the abundance of common zooplankton taxa in the Barents Sea in April 2016.

Variable	Explained Variation, %	F	*p*
Bottom temperature, °C	43	23.69	0.001
Bottom chlorophyll *a*, mg m^−3^	9	5.45	0.001
Mean chlorophyll *a*, mg m^−3^	5	3.47	0.002
Surface chlorophyll *a*, mg m^−3^	6	4.29	0.001
Surface temperature, °C	4	3.6	0.003
Sea ice concentration, %	3	2.67	0.007
Distance from the ice edge, nm	3	2.7	0.006
Mean salinity	2	1.88	0.045
Sampling depth, m	1	1.16	0.332

**Table 6:** Correlation matrix showing the relationship between band axes and significant environmental variables (*p* < 0.05; marked as bold) according to the RDA model in the Barents Sea in April 2016.

Parameter	Axis 1	Axis 2
Eigenvalues	0.549	0.069
Species–environment correlations	0.979	0.855
Cumulative percentage variance of species data	54.9	61.8
Cumulative percentage variance of species–environment relation	71.9	80.1
Bottom temperature, °C	**0.865**	−0.084
Bottom chlorophyll *a*, mg m^−3^	**0.320**	**0.394**
Mean chlorophyll *a*, mg m^−3^	−0.137	0.145
Surface chlorophyll *a*, mg m^−3^	0.108	0.003
Surface temperature, °C	**0.738**	0.015
Ice concentration, %	**−0.354**	−0.129
Distance from the ice edge, nm	−0.074	0.053
Mean salinity	**0.675**	**−0.291**
Sampling depth, m	−0.277	−0.064

**Table 7:** Generalized linear models (GLM) relating abundance of the most frequent zooplankton taxa and total zooplankton abundance to environmental data included in the RDA in the Barents Sea in April 2016. T—temperature (C°), C—chlorophyll *a* concentration (mg m^−3^), S—salinity, C1—surface chlorophyll *a*, mg m^−3^, C2—bottom chlorophyll *a*, mg m^−3^, C3—mean chlorophyll *a*, mg m^−3^, ICE—distance from ice cover (nautical miles), I—ice concentration (%.) Subscripts: 0—surface, bot—bottom. All variables were lg(x + 1)-transformed prior to GLM. Bold font indicates significant models.

Species/Parameter	Factor							
	T0	Tbot	Smean	C1	C2	C3	ICE	I
*Calanus finmarchicus*								
slope	−0.056	−0.045	0.000	**−0.057**	**−0.025**	−0.021	−0.130	0.006
intercept	−0.079	0.408	1.554	**0.243**	**0.076**	0.172	0.884	0.274
*p*	0.793	0.564	0.506	**0.047**	**0.000**	0.273	0.611	0.345
*Calanus glacialis*								
slope	**−0.422**	**−0.179**	**−0.001**	−0.017	−0.006	0.007	0.035	**0.008**
intercept	**0.430**	**0.604**	**1.555**	0.214	0.061	0.145	0.720	**0.268**
*p*	**0.000**	**0.000**	**0.000**	0.323	0.137	0.511	0.806	**0.011**
Copepoda nauplii								
slope	0.388	0.077	−0.0001	**0.081**	−0.005	**0.064**	0.170	0.003
intercept	−0.617	0.270	1.555	**0.090**	0.059	**0.074**	0.553	0.276
*p*	0.048	0.298	0.676	**0.002**	0.495	**0.000**	0.492	0.678
*Metridia longa*								
slope	**−0.430**	**−0.200**	**−0.0005**	−0.018	−0.007	0.003	0.100	0.005
intercept	**0.381**	**0.605**	**1.555**	0.213	0.061	0.150	0.648	0.274
*p*	**0.000**	**0.000**	**0.000**	0.276	0.095	0.773	0.473	0.171
*Microcalanus* spp.								
slope	0.256	0.086	**0.001**	−0.024	−0.013	−0.023	0.364	−0.003
intercept	−0.496	0.245	**1.553**	0.226	0.071	0.187	0.246	0.283
*p*	0.269	0.306	**0.010**	0.468	0.128	0.266	0.185	0.684
*Oithona similis*								
slope	**−0.958**	**−0.346**	**−0.001**	0.016	−0.014	0.015	−0.108	**0.019**
intercept	**2.109**	**1.176**	**1.557**	0.154	0.086	0.119	1.018	**0.236**
*p*	**0.000**	**0.000**	**0.002**	0.674	0.135	0.530	0.739	**0.014**
*Pseudocalanus* spp.								
slope	**−0.819**	**−0.289**	**−0.001**	−0.016	−0.011	−0.003	0.008	**0.014**
intercept	**1.316**	**0.878**	**1.556**	0.220	0.072	0.159	0.752	**0.255**
*p*	**0.000**	**0.000**	**0.003**	0.570	0.119	0.881	0.973	**0.013**
*Oikopleura* juv.								
slope	**0.454**	**0.285**	**0.001**	0.003	−0.003	−0.026	**−0.537**	0.004
intercept	**−0.450**	**0.167**	**1.554**	0.189	0.055	0.172	**1.145**	0.277
*p*	**0.041**	**0.000**	**0.004**	0.925	0.750	0.211	**0.044**	0.599
Total abundance								
slope	**−1.140**	**−0.427**	**−0.001**	0.012	**−0.022**	0.022	−0.021	**0.022**
intercept	**2.872**	**1.491**	**1.557**	0.160	**0.110**	0.095	0.823	**0.220**
*p*	**0.000**	**0.000**	**0.001**	0.792	**0.044**	0.421	0.955	**0.011**

## Data Availability

The data presented in this study are available on request from the corresponding authors (the data are not publicly available due to privacy restrictions).

## Supplementary Materials

The following supporting information can be downloaded at: https://www.mdpi.com/article/10.3390/ani16081213/s1, Table S1. List of sampling stations visited in the Barents Sea in April 2016.
