# Supplementary material for: Spatial Pattern of Spring Mesozooplankton in the Marginal Ice Zone (Northern Barents Sea)
Source: Animals (Basel). 2026 Apr 16;16(8):1213. doi: 10.3390/ani16081213 (PMC13113603; doi:10.3390/ani16081213)
Supplement: Supplementary file 1 [file animals-16-01213-s001.zip › animals-4240384-supplementary.pdf]

Table S1. List of sampling stations visited in the Barents Sea in April 2016.

Transect	ID	Date	Time	Latitude	Longitude	Depth, m	Sampling layer	Ice coverage, %
1	1	13.04.16	9:50	76°26′	34°33′	240	230–0	96
	2	13.04.16	15:00	76°23′	34°34′	288	250–0	93
	3	13.04.16	18:40	76°20′	34°34′	275	260–0	85
	4	13.04.16	23:30	76°0′	34°34′	258	240–0	45
2	5	14.04.16	12:32	76°11′	38°56′	257	230–0	96
	6	14.04.16	16:30	76°10′	39°00′	254	230–0	93
	7	14.04.16	18:00	76°09′	38°59′	263	230–0	90
	8	14.04.16	20:18	76°06′	39°00′	249	240–0	64
	9	14.04.16	23:43	75°45′	39°00′	249	230–0	36
3	10	16.04.16	6:10	78°12′	43°56′	281	260–0	99
	11	16.04.16	12:48	78°10′	43°52′	294	280–0	96
	12	16.04.16	13:27	78°03′	43°52′	296	280–0	92
	13	16.04.16	16:30	78°05′	43°52′	312	300–0	94
	14	16.04.16	22:22	77°43′	43°58′	347	340–0	85
4	15	17.04.16	11:08	78°09′	49°04′	294	290–0	84
	16	19.04.16	16:30	78°41′	49°04′	278	230–0	93
	17	19.04.16	22:25	78°38′	49°04′	273	250–0	94
	18	20.04.16	0:40	78°25′	49°25′	250	230–0	88
	19	20.04.16	2:57	78°14′	49°04′	230	220–0	74
5	20	20.04.16	11:18	78°31′	54°03′	288	280–0	90
	21	20.04.16	16:50	78°31′	54°06′	288	270–0	93
	22	20.04.16	17:55	78°29′	54°04′	266	260–0	92
	23	20.04.16	19:30	78°26′	54°04′	284	270–0	94
	24	20.04.16	22:51	78°15′	54°03′	262	260–0	76
6	25	21.04.16	16:20	78°57′	59°00′	251	230–0	95
	26	24.04.16	6:30	79°00′	58°59′	250	230–0	98
	27	24.04.16	10:30	78°54′	58°57′	273	260–0	90
	28	24.04.16	12:35	78°44′	58°56′	242	220–0	93
	29	24.04.16	18:00	78°33′	59°00′	179	150–0	79
7	30	25.04.16	22:07	79°15′	64°11′	302	280–0	94
	31	26.04.16	5:00	79°09′	64°12′	316	300–0	92
	32	26.04.16	8:00	78°59′	64°09′	359	340–0	93
	33	26.04.16	13:51	78°48′	64°11′	362	350–0	73
